# Supplementary figures and images for: Genome-wide identification of genes involved in beetle odoriferous defensive stink gland function recognizes Laccase2 as the phenoloxidase responsible for toxic para-benzoquinone synthesis
Source: PLoS Genet. 2025 Dec 15;21(12):e1011588. doi: 10.1371/journal.pgen.1011588 (PMC12716784; doi:10.1371/journal.pgen.1011588)

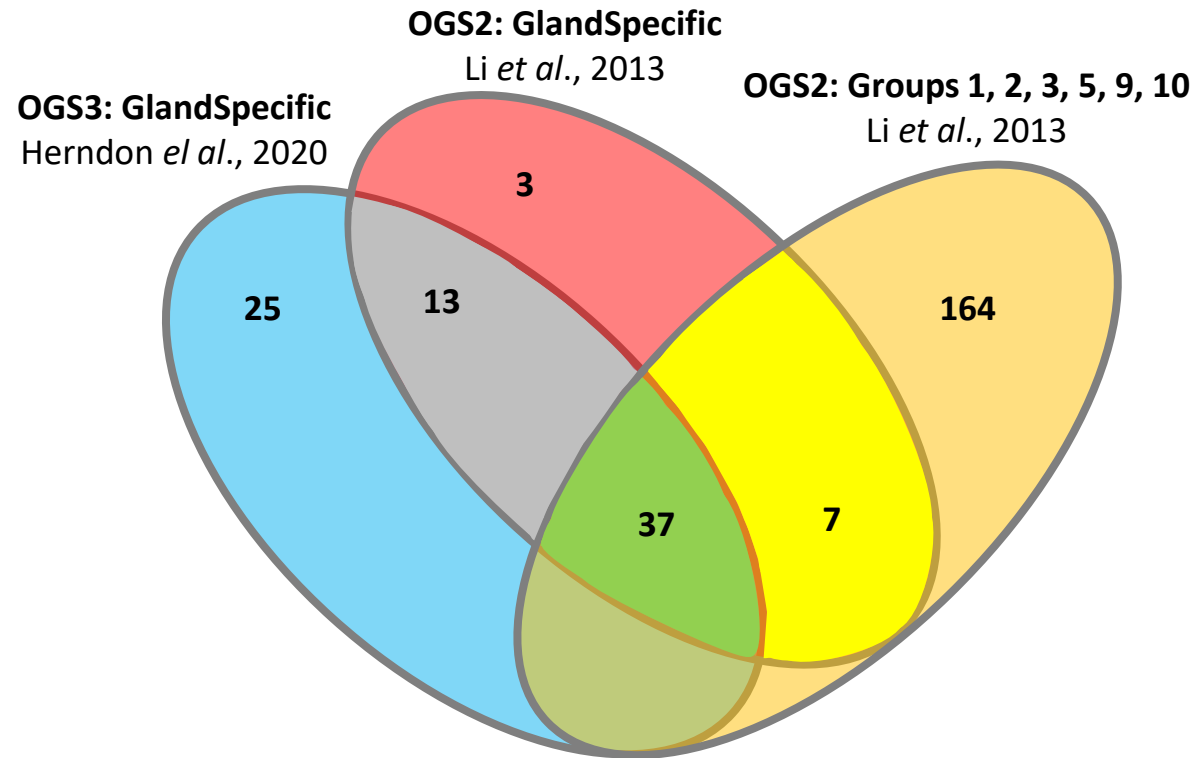

Supplement: S1 Fig — Orange ellipse: 208 stink gland genes identified by Li et al. [38] (OGS2: Groups 1, 2, 3, 5, 9, 10). Blue ellipse: 75 stink gland genes identified by transcriptome re-analysis with OGS3 [34]. Red ellipse: 60 stink gland genes identified by transcriptome re-analysis as performed for OGS3, but with the gene set (OGS2) available at the time of Li et al. [38]. Altogether, the three transcriptomics analyses identified 249 genes with stink gland-specifically enhanced expression (S3 Table). (PDF) [file pgen.1011588.s001.pdf]

## A BlastKOALA: BRITE analysis of KEGG Orthologies

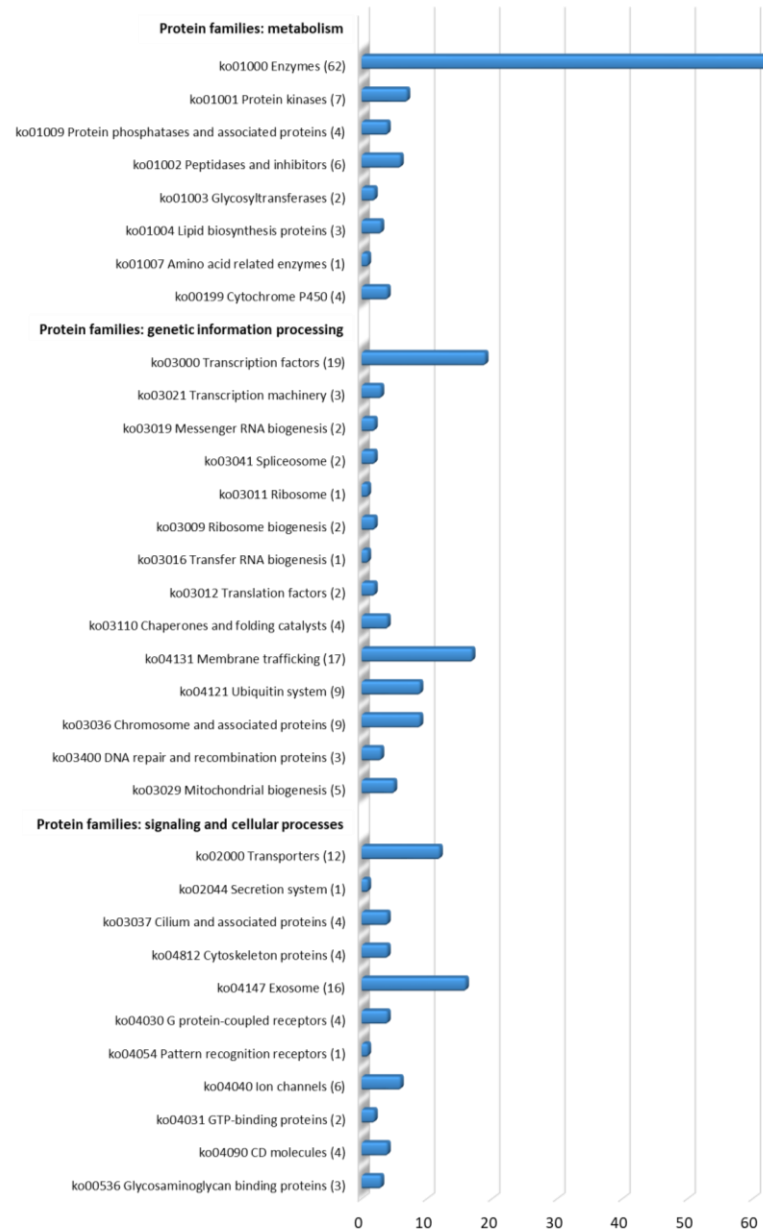

## B eggNOG: BRITE analysis of KEGG Orthologies

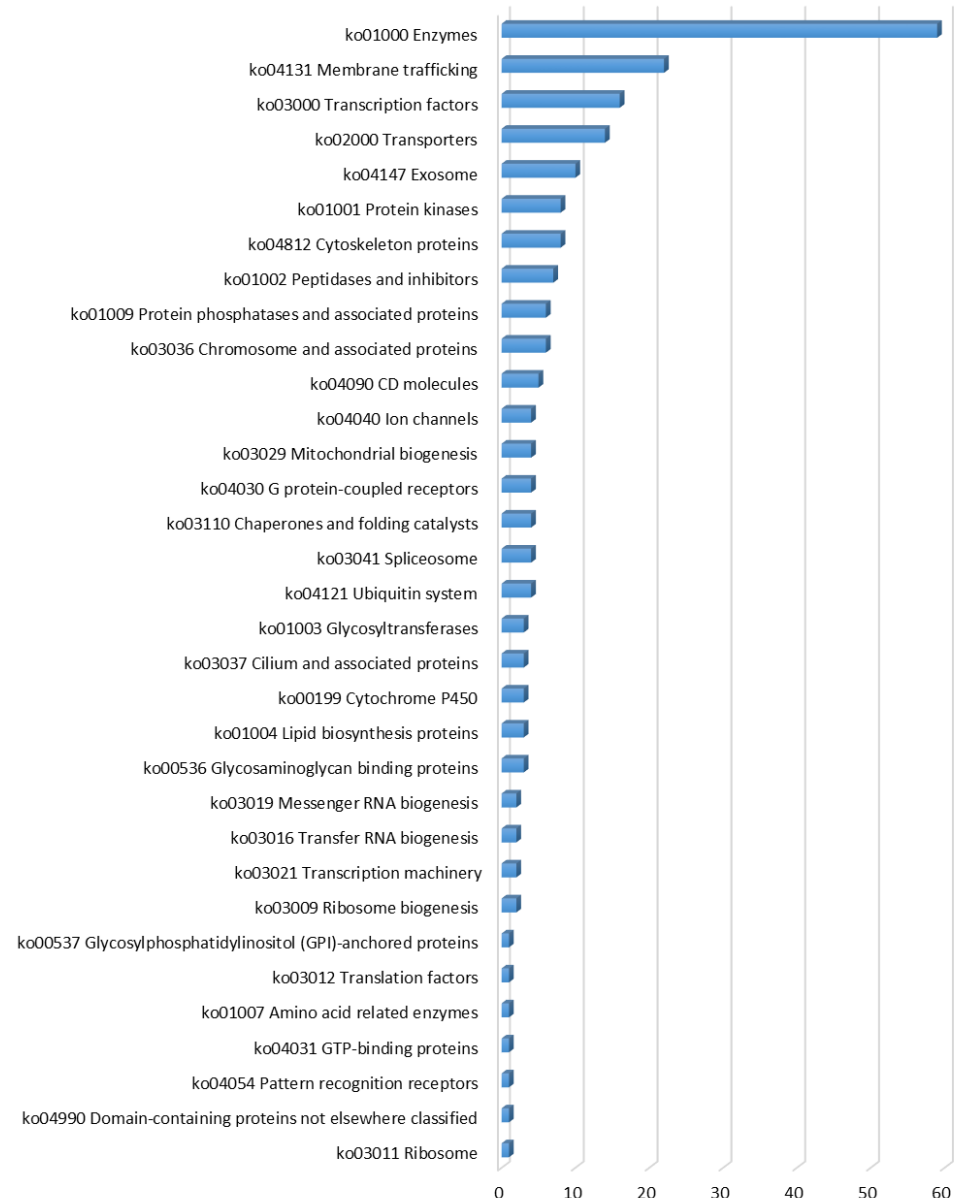

Supplement: S3 Fig — The number of genes that have been assigned to the different KEGG orthology pathways in the Brite analyses of BlastKOALA (A) and eggNOG (B) are presented as column charts. While the BlastKOALA KEGG pathway analysis covered only 198 genes (40%), eggNOG-mapper analyzed 365 genes (75,5%) for the 490 provided query proteins (S6 Table). Similar to the BlastKOALA Brite analysis (A), also the eggNOG-mapper Brite analysis (B) identified many enzymes including kinases, phosphatases, glycosyltransferases, peptidases, and cytochrome P450s as well as transporters and other proteins involved in membrane trafficking. However, also a large number of transcription factors were recognized. (PDF) [file pgen.1011588.s003.pdf]
