# Supplementary material for: Genome-wide identification of genes involved in beetle odoriferous defensive stink gland function recognizes Laccase2 as the phenoloxidase responsible for toxic para-benzoquinone synthesis
Source: PLoS Genet. 2025 Dec 15;21(12):e1011588. doi: 10.1371/journal.pgen.1011588 (PMC12716784; doi:10.1371/journal.pgen.1011588)

A **ShinyGO analysis: significant KEGG pathway SNARE**

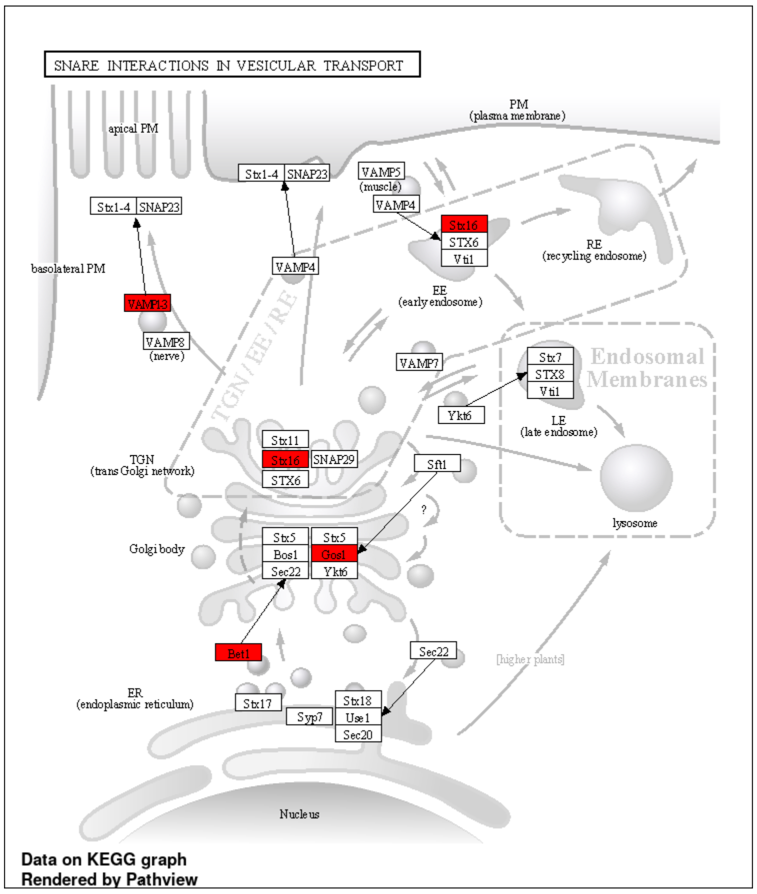

B **BlastKOALA analysis: KEGG pathway LYSOSOME**

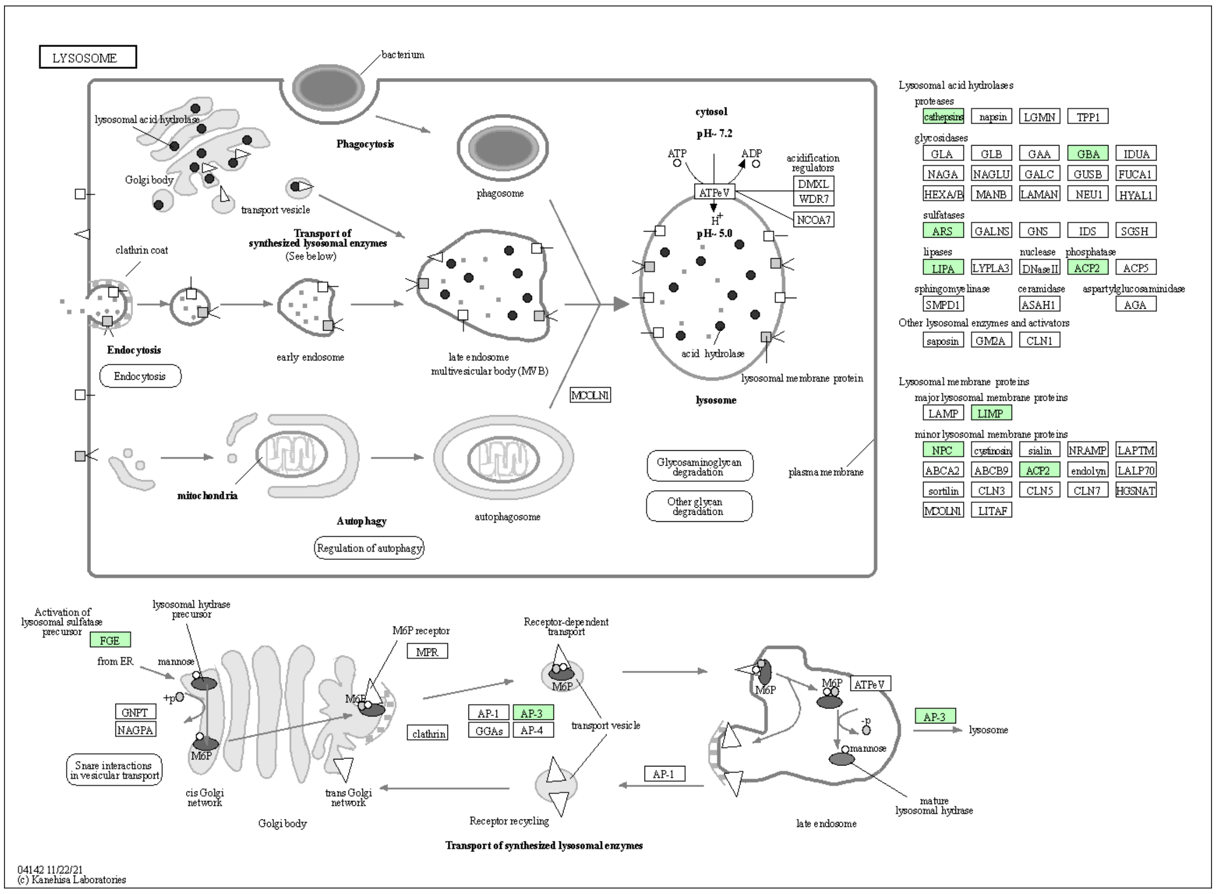

C **BlastKOALA analysis: KEGG pathway PANCREATIC SECRETION**

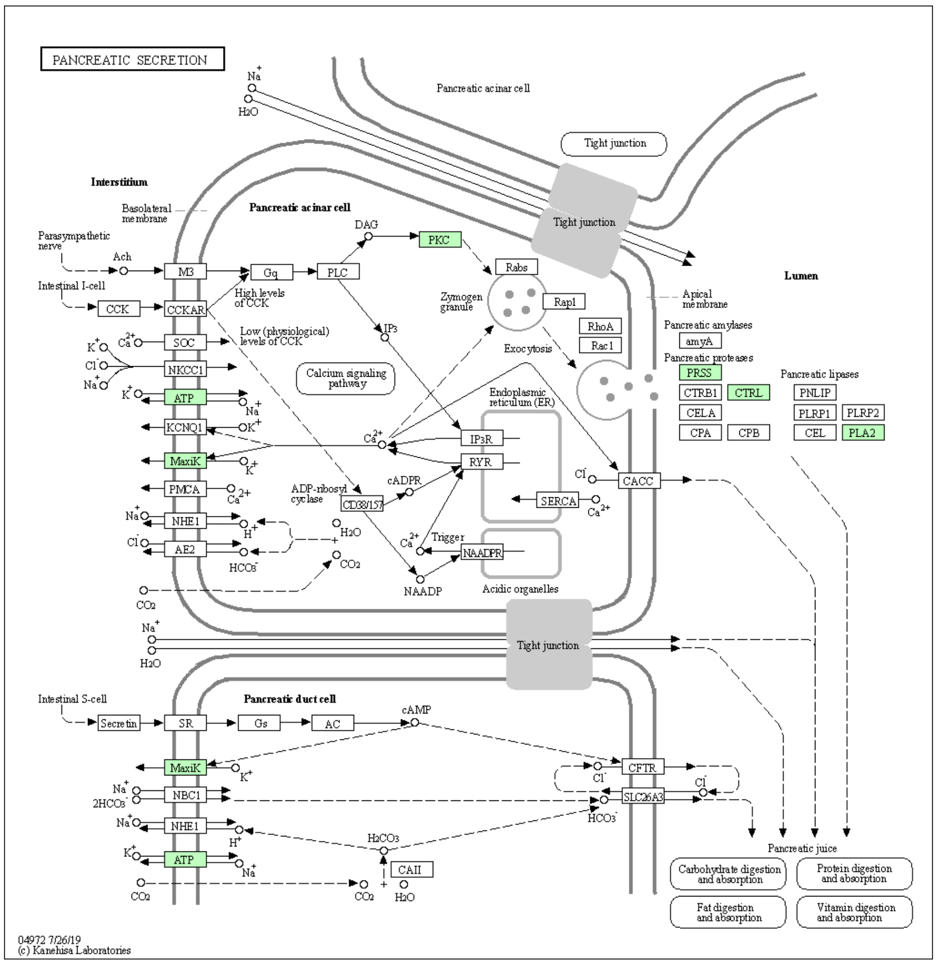

D **BlastKOALA analysis: KEGG pathway MAPK SIGNALING**

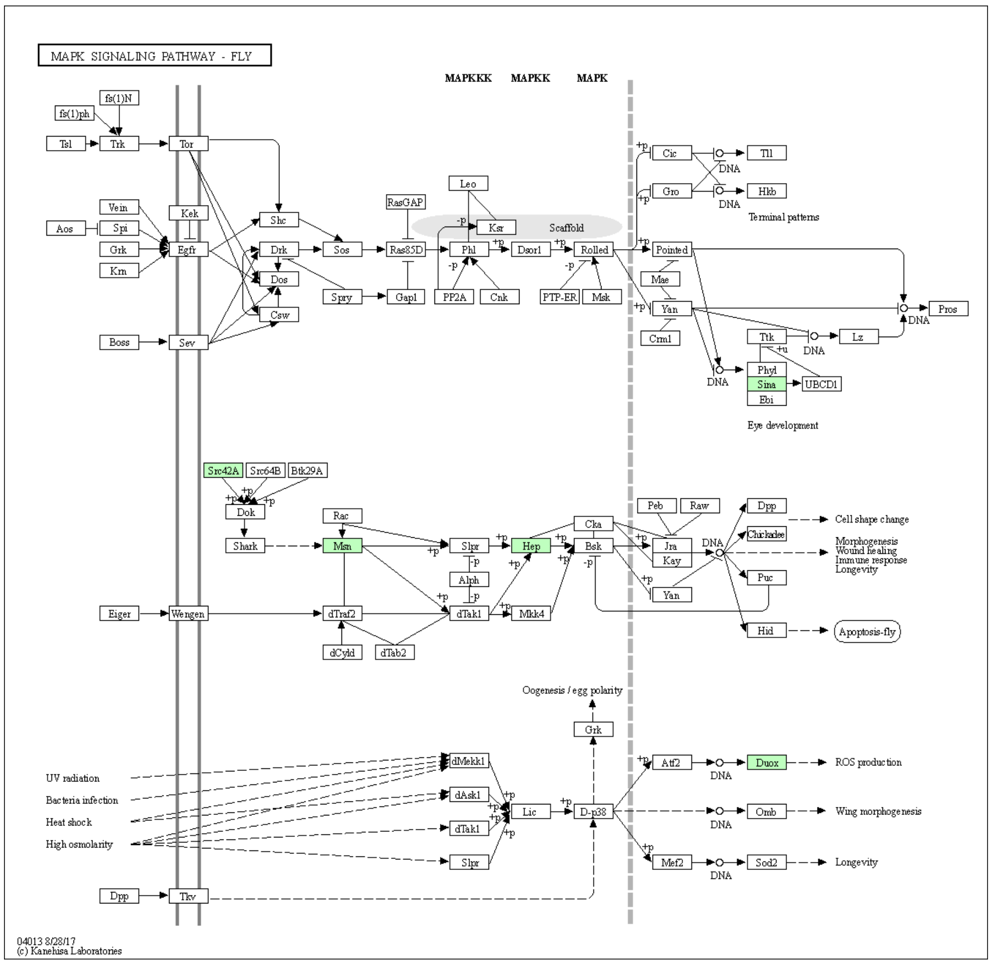

Supplement: S2 Fig — (A) ShinyGO analyzed 438 genes (89%) (S6 Table) and found “SNARE interactions in vesicular transport” as the only significantly enriched KEGG pathway. The five genes Tc_001666, Tc_008320, Tc_009870, Tc_015165, Tc_015183 encoding blocked early in transport 1 (BET1), vesicle-associated membrane protein 7 (VAMP7), syntaxin 16 (STX16), golgi SNAP receptor complex member 1 (GOS1), and vesicle-associated membrane protein 2 (VAMP2), respectively, are all involved in SNARE function (ko04130). In the BlastKOALA analysis, “SNARE interactions in vesicular transport” (ko04130) was identified with the same five genes. In addition (B-D), the following three KEGG pathways were identified: “Lysosome” (ko04142) (B) with 17 genes: Tc_005432 (cathepsin B; CTSB); Tc_008203 (Niemann-Pick C2 protein; NPC2); Tc_008780 (glucosylceramidase; GBA; Tc_008912 (AP-3 complex subunit sigma AP3S); Tc_012600 (octopamine receptor beta; Octbeta); Tc_015151 (arylsulfatase B; ARSB); Tc_015811 (lysosomal acid phosphatase; ACP2); Tc_016314 (formylglycine-generating enzyme FGE; SUMF1); Tc_033512 (G protein-coupled receptor); Tc_034418 and Tc_034419 (TSPAN30; CD63 antigen); as well as Tc_007186, Tc_012838, Tc_012841, and Tc_034670 encoding lysosomal acid lipases/cholesteryl ester hydrolases (LIPA); as well as Tc_014388 and Tc_034847 encoding Major Facilitator Superfamily (MFS) transporters (SLC17A); “Pancreatic secretion” (ko04972) (C) with eight genes: Tc_005635, Tc_007019, Tc_015344, and Tc_030065 (trypsin; PRSS1_2_3); Tc_011288 (potassium large conductance calcium-activated channel subfamily M alpha member 1; KCNMA1); Tc_014033 (sodium/potassium-transporting ATPase subunit beta; ATP1B); Tc_032365 (classical protein kinase C alpha type; PRKCA); Tc_033681 (ovochymase; OVCH); and “MAPK signaling pathway – fly” (ko04013) (D) with six genes: Tc_000385 (mitogen-activated protein kinase kinase 7; MAP2K7); Tc_004593 (dual oxidase; DUOX); Tc_034116 (Runt); Tc_012298 (E3 ubiquitin-protein ligase SIAH1); Tc_014568 [file pgen.1011588.s002.pdf]
