# Supplementary material for: Genome-wide identification of genes involved in beetle odoriferous defensive stink gland function recognizes Laccase2 as the phenoloxidase responsible for toxic para-benzoquinone synthesis
Source: PLoS Genet. 2025 Dec 15;21(12):e1011588. doi: 10.1371/journal.pgen.1011588 (PMC12716784; doi:10.1371/journal.pgen.1011588)
Supplement: Attachment — Submitted filename: Rebuttal Atika_et_al_2025_PLOSgenetics.docx [file pgen.1011588.s012.docx]

**Rebuttal to:**

**Genome-wide identification of genes involved in beetle odoriferous defensive stink gland function recognizes Laccase2 as the phenoloxidase responsible for toxic *para*-benzoquinone synthesis**

Bibi Atika^1¶^, Sabrina Lehmann^1¶^, Elisa Buchberger^1^, Musa Dan’azumi Isah^1^, Zahra Basirkazerouni^1^, Michael Rostás^2^, Gregor Bucher^3^, Ernst A. Wimmer^1*^

**First of all, we would like to thank the editors and reviewers for the careful evaluation of our manuscript. We dealt with all the comments and tried to address the suggestions. This helped us to improve our manuscript. Thank you.**

**Journal Requirements:**

1) We ask that a manuscript source file is provided at Revision. Please upload your manuscript file as a .doc, .docx, .rtf or .tex. If you are providing a .tex file, please upload it under the item type u2018LaTeX Source Fileu2019 and leave your .pdf version as the item type u2018Manuscriptu2019.

**Our submission of the revision complies with the requirements by the journal.**

2) We do not publish any copyright or trademark symbols that usually accompany proprietary names, eg ©, ®, or TM (e.g. next to drug or reagent names). Therefore please remove all instances of trademark/copyright symbols throughout the text, including:

- ® on pages: 19, 20, and 21.

**We removed all copyright and trademark symbols.**

3) Please upload all main figures as separate Figure files in .tif or .eps format. For more information about how to convert and format your figure files please see our guidelines:

<https://journals.plos.org/plosgenetics/s/figures>

**Our submission of the revision complies with the requirements by the journal.**

4) We notice that your supplementary Figures are included in the manuscript file. Please remove them and upload them with the file type 'Supporting Information'. Please ensure that each Supporting Information file has a legend listed in the manuscript after the references list.

**Our submission of the revision complies now with the requirements by the journal.**

5) Some material included in your submission may be copyrighted. According to PLOSu2019s copyright policy, authors who use figures or other material (e.g., graphics, clipart, maps) from another author or copyright holder must demonstrate or obtain permission to publish this material under the Creative Commons Attribution 4.0 International (CC BY 4.0) License used by PLOS journals. Please closely review the details of PLOSu2019s copyright requirements here: PLOS Licenses and Copyright. If you need to request permissions from a copyright holder, you may use PLOS's Copyright Content Permission form.

Please respond directly to this email and provide any known details concerning your material's license terms and permissions required for reuse, even if you have not yet obtained copyright permissions or are unsure of your material's copyright compatibility. Once you have responded and addressed all other outstanding technical requirements, you may resubmit your manuscript within Editorial Manager.

Potential Copyright Issues:

i) Please confirm (a) that you are the photographer of Figures: 1 and 5, or (b) provide written permission from the photographer to publish the photo(s) under our CC BY 4.0 license.

**The first author Bibi Atika is the photographer of the pictures used in Figures 1 and 5. There are no copyright issues regarding this manuscript.**

In particular, as the reviewers point out, reanalyses of the RNAseq data according to the latest TriCast1.1 genome assembly should be completed. Further, integration of more relevant literature and more careful proof-reading would be desirable.

**We have included additional literature as commented by the reviewers.**

**We are very sorry to admit, that we incorrectly referenced Arakane et al. 2005. OLD reference 39 was supposed to** **be Proc Natl Acad Sci U S A. 2005 Aug 9;102(32):11337-42. doi: 10.1073/pnas.0504982102 instead of the one wrongly listed (Insect Mol Biol. 2005;14:453–63).**

**There are several new genome assemblies and there will be again and again. However, no new gene set has been generated and published. Therefore, we used the newest official gene set available (Herndon N, Shelton J, Gerischer L, Ioannidis P, Ninova M, Dönitz J, et al. Enhanced genome assembly and a new official gene set for Tribolium castaneum. BMC Genomics. 2020;21:47), which is also the basis for the iBeetle-screen and iBeetle-Base, which we use for comparison of the phenotypically-derived and the transcriptomics-derived genes.**

**Reviewers' comments:**

**Reviewer #1:** This is a detailed study which reveals interesting and important insights into the function of insect defensive glands. Results of a large systematic study are presented, for which the data appears to be rigorous. I also think it is an important message that 60% of the stink gland genes are not found in Drosophila. For historical reasons Drosophila often acts as a kind of reference insect, and this is another important reason for research such as this, which is extending other insects e.g. Tribolium as model species.

I feel like the clarity and quality of the paper could be improved through some careful rewriting. I found there is quite a lot of detail, particularly in the results part, which could be streamlined, along with some re-writing to make sure the message is always clear and the significance and relation to other studies comes through more strongly. The quality of the writing and use of English could do with improving in quite a few places. For example line 77 ‘secretion’s’, line 79 capitalisation after semicolon, line 187 ‘in view cases’, comma in line 115, line 143 simplify this sentence, line 157-158 the use of English is quite strange. In general I think careful proofreading could improve the paper. There seem to be a couple of inconsistencies such as using Tribolium or T. castaneum, and in gene names e.g. Tc0… or Tc_0…

**We thank the reviewer for spotting all this to help us improve our manuscript.**

- **Line 77 was rephrased and in lines 79 and 80 the capitalization removed**
- **In few cases has been corrected**
- **Comma has been removed**
- **Line 143: Sentence simplified.**
- **Lines 157-158: Sentence rephrased**
- **Tribolium or T. castaneum now consistently used**
- **Tc0 has been replaced by Tc_0 to be consistent in the text of the manuscript.**

I find this point about different classes of genes interesting, and maybe you could discuss this a bit further. i.e. some are specific to the stink gland, whilst some are expressed more widely but still have an important functional role. I see for example that the gene TC030914/ATP7 also has some enriched expression in the free Malpighian tubule according to BeetleAtlas, so maybe there is a common role in secretory cells?

**We added a few more sentences discussing the issue.**

I feel like on line 102 you could do with mentioning e.g. the Brückner, 2021 paper, which provides important insights into a beetle defence gland, and you can highlight how Tribolium can extend or add a useful comparison with what has been found in other insect species. I also feel like some more comparative discussion about findings in other species would be nice at the end of your paper to place your findings in context, and maybe draw out what similarities or differences are emerging.

**The studies on the rove beetle and its tergal gland have been included now into the introduction.**

**We have also added the great work done on rove beetles defensive glands when discussing our findings on laccase 2. Moreover, in the conclusion, we also point out evolutionary implications and differences between the rove beetle situation and the *Tribolium* use of laccase2.**

A couple of small points:

in some places e.g. line 108 you refer to organelle – is this the right term? You seem to be referring to a structure external to the cell? To help with this point, and in general to make your findings clearer it could be nice to have a summary figure labelling the relevant structures and adding the key molecules or pathways.

**‘Vesicular organelle’ is the official term for these cuticle lined parts that lie within the gland cells. We tried to make the sentence clearer in this respect.
In addition, we indicate the vesicular organelles in the schemes of Fig.5E now in red and refer to it specifically in the legend, to clearly identify this important structure.**

Figure 5E – add labels to easily interpret what is being shown in green and blue.

**We added labels and explain that the extracellular green is due to trapping of probe or reactants. Moreover, we also mention the trapping of DAPI in part of the vesicular organelles.**

some places e.g. line 126 it is not clear what you mean by ‘gene models’.

**We replaced gene models by genes, just to not raise any misunderstandings. However, what has been analyzed is the Official Gene Set 3, which relies on “gene models”, that may or may not represent true genes!**

line 114 – make it clear that sclerotization refers to toughening of the cuticle, as PLOS Genetics has a broad readership.

**A short explanation has been added.**

line 287 – can you comment on whether any further genes are found in any of the other new Tribolium castaneum genome versions that have been released?

**There are several new genome assemblies and there will be again and again. However, no new gene set has been generated and published. Therefore, we used the newest official gene set available (Herndon N, Shelton J, Gerischer L, Ioannidis P, Ninova M, Dönitz J, et al. Enhanced genome assembly and a new official gene set for Tribolium castaneum. BMC Genomics. 2020;21:47), which is also the basis for the iBeetle-screen and iBeetle-Base, which we use for comparison of the phenotypically-derived and the transcriptomics-derived genes.**

I assume your raw RNA-Seq data is already deposited to a database, but I wonder whether it would also be useful to deposit you new counts data made against the updated genome?

**The RNA-Seq-Data were deposited already with the publication in 2013. The new counts based on OGS3 are provided completely in Supplementary Table S2, including in comparison also the counts using OGS2.**

**Reviewer #2:** This is a very interesting manuscript describing an RNAi screen and transcriptomics analyses to identify candidate genes that function in the stink gland of the flour beetle Tribolium. The work made use of improved genome assembly and annotations to follow up on their initial screens and identified a reasonable group of genes that may function in development of the glands and in the reactions for producing the protective quinones. The work is thorough and carried out with appropriate experimental designs and statistical analyses.

It is quite interesting that for a large number of the genes of interest, there is no homolog in Drosophila, illustrating a case in which Drosophila is not useful as a model species. The RNAi results combined with chemical analyses demonstrate convincingly that laccase-2 is the oxidase responsible for oxidation of benzophenols to corresponding quinones, and that laccase-2 is localized in the expected tissue and developmental timing. The analysis of the expression and function of splicing isoforms of laccase-2 are also quite interesting. Laccase-2 is not expressed with great specificity in the stink glands because it also functions in cuticle tanning and sclerotization, where it is secreted from epidermal cells after the molt, so serves different oxidative functions.

This is a very well written paper and should be of interest for a wide group, including chemical ecology, biochemistry, and insect molecular biology.

I have suggestions for only a few minor revisions:

It seems to me that Fig 2D is an important part of the results but is presented in a small format that does not highlight its significance. I think it might be presented separately as a more prominent figure.

**This is probably referring to Figure 5D. In the original figure file, the details can be clearly read. We are now providing a high resolution TIFF, in which everything can be deciphered.**

line 317: spelling of lysosome

**Corrected.**

It would be appropriate to cite Arakane et al. "Laccase 2 is the phenoloxidase gene required for beetle cuticle tanning." Proc Natl Acad Sci U S A. 2005 Aug 9;102(32):11337-42. doi: 10.1073/pnas.0504982102 as a key paper on discovery of laccase-2 function in cuticle of Tribolium.

**We are very sorry, that we incorrectly referenced Arakane et al. 2005.
OLD reference 39 was supposed to be Proc Natl Acad Sci U S A. 2005 Aug 9;102(32):11337-42. doi: 10.1073/pnas.0504982102 instead of the one wrongly listed (Insect Mol Biol. 2005;14:453–63). We have corrected this lapsus.**

**Reviewer #3:** Atika et al. presents a comprehensive profiling of gene candidates involved in the production of toxic para-benzoquinones in Tribolium castaneum defensive glands. By reanalyzing a previous transcriptome (Li et al., 2013) and a more extensive genome-wide RNAi screen, they identified a one-third increase in mapping rate, and a two-fold increase in identified genes differentially expressed in the defense gland. Compared to the original study, this work aimed to use improved gene models in the most recent genome annotation, more detailed RNAi phenotypic screen (gland dissection) and relaxed fold-change cutoff, while maintaining the same morphological categories (as in Li et al., 2013) to characterize gland phenotypes as a result of gene-specific RNAi effects. The authors identified concordant and newly annotated genes with stink gland-restricted expression, with a specific highlight of a phenoloxidase Laccase2 in para-benzoquinone synthesis, which was missed in the original transcriptome study.

Overall, I find that the study placed much effort in detailing the gene mining process. The strength of this study lies in the much-needed, updated view of the genes differentially expressed in the stink gland of T. castaneum, which is expected of the past decade of improved convenience of sequencing technologies, alongside significant efforts in phenotypic screens by conducting gland dissections. However, I found that the study falls short of providing some improvements in the transcriptome analysis, and point out several points for improvement in the analysis of transcriptomic data. I also suggest the authors refer to prior work in the literature on laccases in beetles, including in benzoquinone synthesis.

Major comments

1) Overall, this paper does not frame the work sufficiently in a broader biological context, making it quite arduous to read. What is interesting/striking/unexpected about the findings in this paper are not articulated well. It reads instead like a resource paper, devoid of much biological significance. I was also struck that the paper did not mention the detailed recent work on rove beetles that demonstrated a pathway for benzoquinone synthesis that includes a laccase (which again carries out the terminal step). In this case it is not laccase2 but a novel duplicate. See two recent papers: 10.1016/j.cell.2021.11.014, 10.1016/j.cell.2024.05.012 The authors could also discuss the new findings in the context of the previously characterized roles of laccase2 in T. castaneum (10.1073/pnas.0504982102), which appears to be conserved in insects. There are no evolutionary inferences in this paper to do with the co-option/pleiotropy of laccase2. The authors need to frame the findings of the paper in the context of these studies.

**We have added the great work done on rove beetles defensive glands and discuss our findings in respect to this work. In the conclusion, we also point out evolutionary implications and differences between the rove beetle situation and the Tribolium use of laccase2.**

2) The phenotypic characterization does not seem intuitive and requires revision. Comparisons of different categories are not very clear. I suggest adding scale bars, especially to clarify size differences that are not obvious in III and VII. Also, comparison of colors would maybe require similar lighting contrast in all panels, which is not the case at least in VI (too light) and VII (too dark).

In Figure 1,

- III appears slightly damaged and irregularity in size is not immediately obvious. If possible I suggest replacing this example.

**These images were taken during a large screen analyzing thousands of genes on different days and different stereomicroscopes. These are examples of phenotypes that were put in the respective category. These phenotypes are clearly not absolutely distinct and lighter to colorless as well as darker to melanized are to some degree subjective. In addition, depending on the exact day of analysis the phenotypes might actually slightly differ. This, however, is typical for a large screen and is hard to rectify afterwards. The image for III has been replaced and the contrast has been adjusted.**

**We refrained from adding a scale bar, since we have an internal size reference: in all the pictures the last abdominal segment is depicted along with the glands. The last abdominal segment has a width of 0.7 to 0.8 mm, which is now also indicated in the figure legend. This allows to compare glands even in the case the adult beetles were slightly different in size and therefore serves as a better comparison than a scale bar.**

3) Reanalysis of transcriptome data should include the log adjusted p-values for including significance values. From their reanalysis of transcriptome data, they reduced the log2 fold cutoff from 8 to 4 and highlighted that it allowed them to identify lac2. While it is expected that reducing the cutoff can result in fewer false negatives, especially given the not-so-well mapped sequencing data, presenting the data in terms of both log2 fold change and p-value significance, in the form of a volcano plot will be a better way forward.

**We agree that volcano plots would be ideal to present the significance of the identified genes. However, we cannot provide this. The RNAseq data were generated in 2011 and published in 2013, at a time sequencing was not as cheap as today and when the standards were not established yet. Therefore, all the different samples were only sequenced once. We could do complicated statistics, which is in my view meaningless with N=1 for the control. To reduce the false positives, we only considered genes that were 16x higher expressed in all four gland tissues compared to the control. In our opinion, this is actually much more restrictive than the approach in Li et al 2013, were only two tissues were compared to identify genes. Therefore, we also identified much less genes as Li et al. with 161 genes from the Li et al paper not being recognized by our new approach. Laccase two actually was identified in the phenotypic screen and would have not been picked up in the transcriptomics due to our cut off limit (being 16x higher only in three out of the four tissue samples).**

3) Gene ontology analyses are not very informative. I don’t find Figure 4 particularly informative, especially with “Human Diseases” as a functional category in this context. I suggest that the authors proceed with more caution and reduce descriptive emphasis using gene ontology and kegg pathway analyses, instead of using them as factual descriptions of underlying biology in transcriptomic data.

**We do agree with the reviewer, as we also find GO analyses rather meaningless and boring. However, such papers need to provide these data especially for meta-analyses. Moreover, some of the GO data can be meaningful. To reduce its emphasis, we have shortened this section in the main text by moving the details to the respective supplementary figure legends.**

4) Isoform-specific roles of laccase2 in benzoquinone synthesis The authors reported that “when only the minor splice variant (Tc_Lac2B) was targeted, the stink glands appeared partially melanized and the volatile para-benzoquinones were only reduced” (line 385), then went on to mention that Tc_Lac2B “contributes little to cuticle tanning but was nonetheless indispensable for survival”. I found this to have digressed from the original point on the roles of laccases in gland-specific functions, and instead recommend the authors to address the melanized phenotype of gland secretion.

**To be honest, we have no good idea, what the “melanized” phenotype really means. Only for the morphological phenotype colorless there seems to be a correlation with reduced quinones. For all other morphological phenotypes, no direct correlation between the morphological phenotype and the volatile compound phenotype could be recognized (Lehmann et al., 2022). What happens exactly in the melanized phenotypes, we don’t know. Whether melanin actually is involved as a immune system response or whether the dark color comes from crystallization of specific components, we do not know and might be actually different for different genes. The reference to the cuticle tanning is only to indicate that also here LacA causes the major phenotype while LacB has a minor function, for which we say: “both isoforms are needed for proper biosynthesis of secretion, with Tc_Lac2A being necessary for the oxidation of para-benzoquinone precursors and an unclear role for Tc_Lac2B.” Sorry, I do not know, what we can say more in the moment for the specific function of LacB.**

In addition, I urge the authors to validate using RT-PCR the RNAi knockdown of isoform-specific laccase2, especially with previously reported work that showed unintended reduction of Lac2A with dsLac2B (Fig 8A in 10.1073/pnas.0504982102) despite exon-specific design of dsRNA for each isoform.

**In Arakane et al. 2005, the authors used RT-PCR, which is limited in quantification and stated in the manuscript: “The knockdown levels of the transcripts of TcLac2A and TcLac2B were examined by RT-PCR (Fig. 8A, which is published as supporting information on the PNAS web site). Injection of dsLac2A reduced the TcLac2A transcript level without reducing that of TcLac2B. Conversely, the introduction of dsLac2B reduced the TcLac2B transcript level without affecting that of TcLac2A. Thus, there seems no indication of cross-gene knock-down. Moreover, RNAi is most of the times only a knock-down anyway, and even some minor cross-acting effect would not change the interpretation of the data.**

Minor comments

Line 188: “In the case” seems redundant

**Rephrased.**

Line 301: omit “for”

**Rephrased.**

Line 317: “Lysosome” spelt incorrectly

**Corrected.**

Line 352: “but with the value 3,1 not in the female abdominal sample”

**Rephrased.**

Line 520: “placed” instead of “places”

**Corrected.**

Line 522: “added” instead of “put”

**Corrected.**

Page 28: Fig 2 has “Abdominal” consistently misspelt

**Corrected.**

Rewording the title of Figure 3 will be more informative for the reader

**Figure Title was reworded:
Phenotypic screen iBeetle and different transcriptomics analyses recognize 490 gland genes with limited overlap.**

Figure 5: Changing the color scheme to complementary colors like azure and orange, or magenta and green will provide better contrast.

**We changed the color scheme.**

- I believe the genes are incorrectly referred to as TC0101490 and TC0101489 in the Figure legend. There are some inconsistencies in gene naming and numbering, and I suggest a review of all such cases.

**Thank you for picking this up. We corrected this in the beginning of the Figure legend and also corrected the mistakes and different styles of numbering.**

- It will be helpful for the reader if the authors can use dotted lines to demarcate the boundaries of individual cells in panel E. Could the authors also provide an explanation for the fluorescence observed in cell type 2 panel, for Lac2AB RNAi?

**We added dotted lines and explain that the extracellular green is due to trapping of probe or reactants. Moreover, we also mention the trapping of DAPI in part of the vesicular organelles.**

There are many other typos throughout the manuscript, but I did not note them all.

**We tried to pick up on typos and corrected them.**
